# Associations Between Single-Family Room Care and Breastfeeding Rates in Preterm Infants

**DOI:** 10.1177/0890334420962709

**Published:** 2020-10-09

**Authors:** Hege Grundt, Bente Silnes Tandberg, Renée Flacking, Jorunn Drageset, Atle Moen

**Affiliations:** 1 60498 Department of Neonatology, Haukeland University Hospital; 2 155273 Department of Pediatric and Adolescent Medicine, Drammen Hospital, Vestre Viken Hospital Trust; 3 Lovisenberg Diaconal University College; 4 Department of Clinical Science, Faculty of Medicine and Dentistry, University of Bergen; 5 3317 School of Education, Health and Social Studies, Dalarna University, Sweden; 6 1658 Department of Global Public Health and Primary Care, Faculty of Medicine, University of Bergen; 7 Institute of Nursing, Faculty of Health and Social sciences, Western Norway University of Applied Sciences, Bergen, Norway; 8 Department of Neonatology, Oslo University Hospital

**Keywords:** breastfeeding, Breastfeeding Self-Efficacy Scale–Short Form, family-centered care, milk supply, mothers milk, neonatal intensive care unit design, prematurity, pumping, single-family room

## Abstract

**Background:**

Hospitalization in neonatal intensive care units with a single-family room design enables continuous maternal presence, but less is known regarding the association with milk production and breastfeeding.

**Research aim:**

To compare maternal milk production, breastfeeding self-efficacy, the extent to which infants received mother’s milk, and rate of direct breastfeeding in a single-family room to an open bay neonatal intensive care unit.

**Methods:**

A longitudinal, prospective observational study comparing 77 infants born at 28– 32° weeks gestational age and their 66 mothers (*n* = 35 infants of *n* = 30 mothers in single family room and *n* = 42 infants of *n* = 36 mothers in open bay). Comparisons were made on milk volume produced, the extent to which infants were fed mother’s milk, and rate of direct breastfeeding from birth to 4 months’ corrected infant age. Breastfeeding self-efficacy was compared across mothers who directly breastfed at discharge (*n* = 45).

**Results:**

First expression (6 hr vs. 30 hr, *p* < .001) and first attempt at breastfeeding (48 hr vs. 109 hr, *p* < .001) occurred significantly earlier, infants were fed a greater amount of mother’s milk (*p* < .04), and significantly more infants having single-family room care were exclusively directly breastfed from discharge until 4 months’ corrected age; *OR* 6.8 (95% CI [2.4, 19.1]). Volumes of milk produced and breastfeeding self-efficacy did not differ significantly between participants in either units.

**Conclusion:**

To increase the extent to which infants are fed mother’s own milk and are exclusively directly breastfed, the design of neonatal intensive care units should facilitate continuous maternal presence and privacy for the mother–infant dyad.

## Abstract (es)

**Bakgrunn for studien:**

Nyfødtavdelinger som er tilrettelagt med familierom muliggjør at mødre kan bo med spedbarnet hele innleggelsen. Kunnskapsgrunnlaget om hvilken innflytelse dette har på melkeproduksjon og direkte amming er svakt.

**Hensikt:**

Sammenligne melkeproduksjon, mestringsforventning til amming, grad av morsmelkernæring og direkte amming i en nyfødtavdeling tilrettelagt med familierom til alle spedbarn sammenlignet med i en nyfødtavdeling der alle spedbarn ligger i flersengstuer.

**Metode:**

En prospektiv observasjonsstudie som sammenligner 77 spedbarn født ved 28– 32° ukers gestasjonsalder og deres 66 mødre (*n* = 35/30 i familieromsavdeling og 42/36 i flersengstueavdeling). Sammenligning ble gjort på produserte melkevolum på dag syv, 14 og ved spedbarnets gestasjonsalder 34°, og på grad av morsmelkernæring og direkte amming fra fødsel til fire måneders korrigert alder. Mestringsforventning til amming ble sammenlignet mellom mødre som ammet direkte ved utreise fra avdelingen (n= 45).

**Resultat:**

Mødre håndmelket seg første gang tidligere (median 6 timer versus 30, *p* <.001), hadde første direkte ammeforsøk tidligere (median 48 timer versus 109, *p* <.001) og spedbarn ble matet med morsmelk i større grad i familieromsavdelingen sammenlignet med flersengstueavdelingen (*p* < .04). I tillegg ble signifikant flere spedbarn eksklusivt direkte ammet til fire måneders korrigert alder i familieromsavdelingen (OR 6.8 (95% CI: [2.4, 19.1]). Det var ingen signifikante forskjeller i produserte melkevolum eller mestringsforventning til amming.

**Konklusjon:**

For å øke graden av morsmelkernæring og direkte amming, bør nyfødtavdelinger tilrettelegge for at mødre kan bo sammen med spedbarnet hele innleggelsen, og gi mor-barn dyaden mulighet for privatliv.

**Editor’s note:**

This abstract was verified through a back translation by Roger Mathisen, MSc

## Background

Mother’s own milk provides substantial health benefits to preterm infants (Dieterich et al., 2013). To provide milk and breastfeed can be perceived as highly meaningful and strengthen the mother–infant relationship during hospitalization in a neonatal intensive care unit (NICU) (Flacking et al., 2016). However, to maintain milk expression for weeks after a preterm birth has been reported as emotionally challenging (Bujold et al., 2018), and associated with lower success in producing adequate volumes of milk and establishing direct breastfeeding (Wilson et al., 2018). Preterm infants often receive little to none of their nutritional intake from their mother’s own milk, and breastfeeding rates vary widely (between 19%–70%) at discharge in European NICUs (Bonet et al., 2010). Direct breastfeeding can be challenging, and affected by factors in the infant (i.e., immaturity, gestational age, morbidity, male gender, or multiples), the mother (i.e., psychological well-being, motivation, self-efficacy, level of education, or smoking), NICU care practices (i.e., the use of skin-to-skin care, nipple shields, or pacifiers; Maastrup et al., 2014), and architectural design (van Veenendaal et al., 2019).

Maternal perceived expectation of their own ability to cope with breastfeeding is commonly referred to as breastfeeding self-efficacy (BSE), and may influence the effort a mother undertakes to succeed in breastfeeding. A higher level of BSE has been associated with greater success in breastfeeding (Dennis & Faux, 1999). BSE may be influenced through interactions with the infant, previous maternal experience with performance and behavior, observation of others successfully breastfeeding, receiving breastfeeding encouragement, and maternal health. Interventions aimed at improving BSE have been found to be an effective way to increase breastfeeding rates at 1 and 2 months postpartum in healthy term infants (Brockway et al., 2017).

**Key Message**

- A lack of studies on the associations between single-family room care and milk expression and breastfeeding in mothers of preterm infants exists.
- Single-family room care was associated with earlier timing of first milk expression and attempts at breastfeeding, and infants fed mother’s milk and sustained exclusively directly breastfeeding up until 4 months corrected age to a greater extent when compared to open bay unit care.
- To increase preterm infants receiving mother’s milk and exclusively directly breastfeeding, the design of neonatal intensive care units should facilitate both continuous presence of the mother and privacy for the mother–infant dyad.

Traditional open bay (OB) NICUs reduce maternal presence and involvement in care by lack of shared accommodation and hospital regulations restricting parental presence. In contrast, a single family room (SFR) NICU design facilitates continuous parental presence, reduces stressful stimuli, facilitates privacy, and allows undisturbed parent–infant closeness with longer periods of skin-to skin care (SSC; Dunn et al., 2016). We have previously reported large differences in duration of parental presence and SSC between OB and SFR NICU care (Tandberg, Frøslie et al., 2019), with concurrent reduction in depression scores among SFR mothers and reduced stress in both parents (Tandberg, Flacking et al., 2019). SSC facilitates milk production and direct breastfeeding, and is associated with improved breastfeeding rates among preterm infants (Sharma et al., 2019). Vohr et al. (2017) found that SFR designs increased the volumes of expressed mother’s milk. However, there also have been reports of SFR units which had no influence on the volume of expressed mother’s milk (Dowling et al., 2012). In Sweden, where parents have unrestricted access to NICUs and many units have SFRs, the breastfeeding prevalence in preterm infants fell significantly over a 10-year period despite a potential increase in parental involvement and presence (Ericson et al., 2016).

There is a lack of knowledge regarding the association between SFR design and maternal milk production and breastfeeding. We aimed to compare maternal milk production, breastfeeding self-efficacy, the extent to which infants received mother’s milk, and rate of direct breastfeeding in a SFR to an OB NICU.

## Methods

### Design

This study had a longitudinal, prospective, comparative, observational design and was approved by the Norwegian Regional Committee for Medical Research Ethics (REK no. 2013/1076). The rationale for this design is that all families in the SFR unit had the right to be continuously present and share accommodation with the infant throughout hospitalization. It would have been practically and ethically impossible not to provide this level of care to a control group. A comparative study design is supported in a Cochrane review (Shields et al., 2012) that assessed family-centered care for hospitalized children. The reviewers stated an urgent need for more research to evaluate the effect of family-centered care implementation in hospitals and argued that a comparison between different hospitals can provide opportunities for a sound evaluation. This study was part of a larger study comparing the two units.

### Setting

One SFR unit and one OB unit in two different hospital catchment areas more than 400 km (249 mi) apart participated in the study. Both units provided care until discharge at maternity hospitals, and encouraged both parents to be present as much as possible. Parents received full economic compensation for loss of income during the hospital stay.

In the SFR unit, every family had adjustable and comfortable hospital beds in their own private room, with full overnight accommodation for both parents and facilities for the preterm infant. SSC was encouraged by the staff. The unit provided full meals to both parents, and siblings were welcome to stay. There was a breast pump in every room.

The OB unit had several open bays facing a corridor. Four to eight infants shared one room throughout hospitalization. Parents had unrestricted access to the unit, with exceptions made during medical rounds and some medical procedures. As the delivery unit was located in a different building approximately 500 m (0.3 mi) from the NICU, transport was by ambulance at admittance and for visitation by hospitalized mothers. Basic overnight accommodation outside the NICU building was available. Rooming-in was possible during the last days prior to discharge in a room outside of the unit. SSC was encouraged, facilitated with comfortable recliners beside the cot or incubator. Privacy was attained with moveable floor screens. The unit provided all meals for one parent, and siblings could visit with limitations. Three breast pumps within and two outside the unit were available for mothers to share. Breast pumps rented through the pharmacy department were reimbursed.

Both units actively promoted expressing milk and breastfeeding from Day 1, with all nurses trained to guide milk production and direct breastfeeding. The SFR unit had five fully-trained lactation support providers, the OB unit had six. Mothers were advised to express by hand 6–8 times per day in the first 2 days after birth, and thereafter to double pump by electric breast pump at least 6–8 times per day, including once during the night. The same brand of electric breast pump was used in both NICUs.

As part of the study the units agreed on a common feeding protocol. Enteral feeds were begun using either donor milk or preterm formula if the mother’s own milk was not available. This was then replaced with the mother’s own milk as production increased.

### Sample

**Figure 1:**
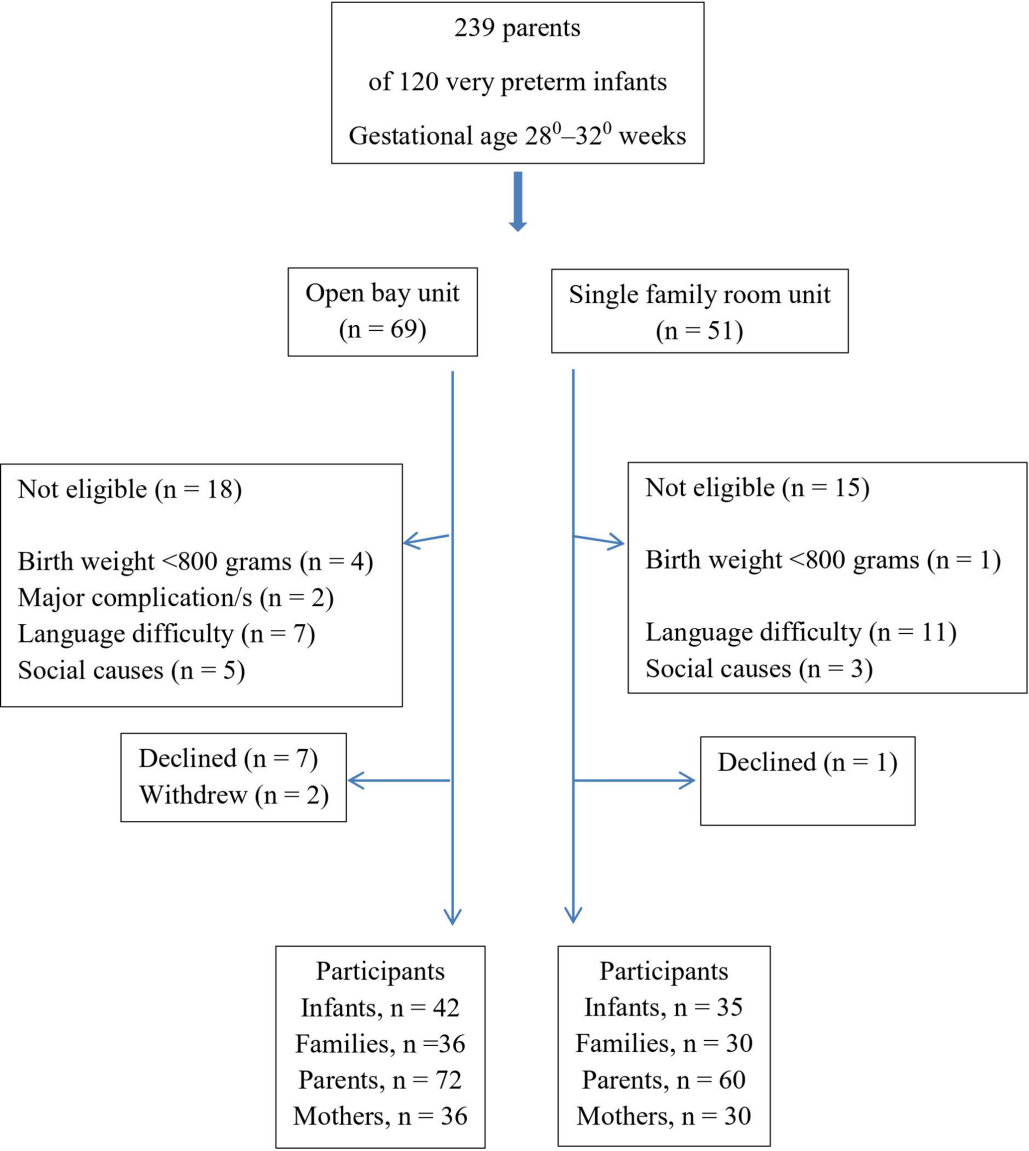
Flowchart.

We included consecutive mothers with infants born at a gestational age (GA) of 28–32° weeks. Exclusion criteria were: parents not speaking Norwegian, the infant being in the custody of the child protection service, drug-abusing mother, one parent suffering from a mental illness, birthweight < 800 g, triplets/quadruplets, and infants with severe morbidities. From the eligible cohort (120 infants), 77 infants (SFR *n* = 35, OB *n* = 42) and their 66 mothers (SFR *n* = 30, OB *n* = 36) were enrolled in the study (Figure 1). The power calculation was based on the main outcome in the main study (Tandberg, Frøslie et al., 2019, Tandberg, Flacking et al., 2019).

### Measurements

We compared first time mothers expressed milk and attempted direct breastfeeding (hours postpartum), the number of breastfeeding attempts from post menstrual age (PMA) 32–34°, and the total volume of milk expressed and/or directly breastfed at Days 7, 14, and at PMA 34° weeks, reported as mL per 24 hr. Directly breastfed volumes were measured with test weighing; 1 g of infant weight gain was considered equivalent to 1 mL of milk.

At PMA 32°, 33°, and 34°, and at discharge, term date, and 4 months’ corrected age, we compared the extent to which infants were fed mother’s own milk and/or donor human milk, categorized in accordance with the World Health Organization’s (WHO, 2019) classifications as exclusively (drops, syrups, medicine), fully (liquids, drops, syrups, but not non-human milk or food based fluids), partly (mother’s milk supplemented with other nutrition), or formula-fed. We also compared how infants were fed at discharge, term date, and at 4 months corrected age, categorized as exclusively directly breastfed (fed only from the breast), partly directly breastfed (fed from the breast and by gavage/cup/spoon/bottle), or not directly breastfed. The use of nipple shields during breastfeeding was compared at PMA 32°, 33°, 34°, discharge, term date and 4 months’ corrected age.

Participants who breastfed directly (exclusively or partly) answered the *Breastfeeding Self-Efficacy Scale–Short Form* (BSES–SF) questionnaire (see Supplementary Material) at discharge (Dennis & Faux, 1999). BSES–SF addresses the mothers’ perceived technical skills and subjective feelings about breastfeeding through 14 statements (“I can always determine that my baby is getting enough milk”), rated on a 5-point Likert scale, possible range from 14 to 70. A higher score indicates a higher self-efficacy, associated with greater success in breastfeeding (Dennis & Faux, 1999). The questionnaire has been translated into Norwegian (Haga, 2012) and found reliable and valid in preterm and ill newborns (Tuthill et al., 2016). A reliability analysis was carried out in our population on the BSES–SF scale comprising 14 items. Cronbach’s alpha showed the questionnaire to reach high reliability, α = 0.927. All items appeared to be worthy of retention, resulting in alpha remaining unchanged or declining if deleted.

### Data Collection

Participants were recruited consecutively from May 1, 2014 until July 31, 2016. A designated research nurse at each unit approached all parents of infants who met the inclusion criteria, within 2 days postpartum. Oral and written information was provided before signed informed consent was retrieved from both parents upon recruitment. Participants’ confidentiality was maintained throughout the study by the use of personal identification numbers. Identifying keys and data were stored in secure research servers at the participating hospitals. The data have been stored at the respective research servers according to the requirements of the hospitals and the ethical committee. Data were reported by participants or retrieved from the medical charts by the designated research nurses. Study team verification was conducted to the extent possible. At term date and 4 months corrected age the infants and parents returned to the units and data were reported for these time points.

### Data Analysis

Demographic variables, milk volumes, BSES–SF, the extent to which infants received mother’s milk and rate of direct breastfeeding were first compared by bivariate analyses; two-sample *t*-tests, and Pearson’s chi-square tests. Descriptive statistics are given as means (*M*) with standard deviation (*SD*), medians (*Mdn*) with quartiles (*q*1*–q*3) or frequencies (%) according to the type and distribution of data. Due to the correlation structure within the repeated measurements, differences in outcomes were further analyzed using mixed models. These multilevel models regard the repeated measures as Level 1 data and the participants as Level 2 data, thereby dealing with the autocorrelation across the repeated measurements. Variables were controlled for time, mode of delivery, maternal education, twin or not, gestational age at birth, and hospital care (Single-Family Room/Open Bay NICUs). For the continuous outcome variable (volume of mother’s milk) the mixed model was a multiple linear regression model, results given as *B* coefficients (to be interpreted as the mean difference between the SFR and OB units, controlled for the model covariates). For the categorical outcome variables (the extent to which infants received mother’s milk and direct breastfeeding) a multiple logistic mixed model regression analysis was performed, results presented as odds ratios (*OR*) with corresponding 95% CI (to be interpreted as the ratio of the odds of the outcomes in question occurring in participant infants with SFR care to the odds of it occurring in participant infants with OB care, controlled for the model covariates). It is a measure of the strength of the association between SFR exposure and the outcomes. Data were analyzed using SPSS (Version 24, IBM 2010). A *p*-value < .05 was considered statistically significant.

## Results

The study groups were similar except for a significantly lower GA in the OB unit (*p* = .03). Mother participants in the SFR unit were present more in the NICU throughout hospitalization (*p* = < .001). They also gave more SSC per day (*p* = .002; Table 1). Infant participants in the SFR unit were more often delivered by caesarean (*p* = .04) and SFR participant mothers had a lower level of education (*p* = .02). Participant infants in the OB unit were more often initially mechanically ventilated (*p* = .01), but time on mechanical ventilation was short (usually a few hours). In general, morbidity was low, and no clinical differences were seen between the groups (Table 2).

**Table 1:** Comparison of Characteristics of Infants (*N* = 77) and Mothers (*N*= 66) Grouped by Type of Hospital Care.

Characteristic	Type of Hospital Care				*t*	*p*
	SFR *n* = 35 (45.5%) Infants *n* = 30 (45.5%) Mothers		OB *n* = 42 (54.5%) Infants *n* = 36 (54.5%) Mothers			
	*M (SD*)	Min-max	*M (SD*)	Min-max		
Infants						
GA at birth (weeks ^days^)	30^5^ (1)	28^2^–32^0^	30^1^ (1)	28^1^–31^6^	2.48	.03
PMA at discharge (days)	252 (9)	232–270	255 (14)	242–332	-1.05	.27
Length of stay (days)	38 (12)	22–61	44 (18)	25–134	-1.62	.16
Birthweight (g)	1452 (301)	910–2134	1382 (274)	945–2055	1.06	.29
Discharge weight (g)	2271 (299)	1840–2830	2317 (297)	1700–3318	-.67	.51
Mothers						
Age (years)	31 (7)	19–47	32 (6)	21–44	-.88	.38
Presence in the unit (hr/day)	21 (7)	11–24	7 (3)	1–12	32.5	< .001
Skin to skin care (hr/day)	4 (2)	.1–9	3 (2)	.1–10	1.33	.002

*Note*. The table presents infant and maternal characteristics at a continuous level. SFR = single-family room unit; OB = open bay unit; GA = gestational age; PMA = postmenstrual age.

**Table 2:** Comparison of Characteristics of Infants (*n* = 77) and Mothers (*n* = 66) Grouped by Type of Hospital Care.

Characteristics	Hospital Care		*Χ* ^2^	*P*
	SFR *n* (%)	OB *n* (%)		
Infants	35 (45.5%)	42 (54.5%)		
Gender (male)	16 (46)	27 (64)	2.67	.01
Twins	10 (29)	18 (43)	1.06	.30
Cesarean birth	25 (71)	20 (48)	4.46	.04
Mechanical ventilation	0 (0)	9 (21)	10.69	.01
Mothers	*n* = 30 (45.5%)	*n* = 36 (54.5%)		
Norwegian first language	24 (80)	30 (83)	4.54	.21
Smoking	1 (3)	0 (0)	0.01	.92
Married/cohabitant	30 (100)	33 (92)	1.38	.64
Care responsibility for siblings at home	8 (23)	11 (26)	0.11	.74
Level of education			10.40	.02
Elementary school	4 (13)	0 (0)		
High school	10 (33)	10 (28)		
College/university	15 (50)	24 (67)		

*Note*. The table presents infant and maternal characteristics at ordinal or nominal level. SFR = single-family room unit; OB = open bay unit.

**Figure 2:**
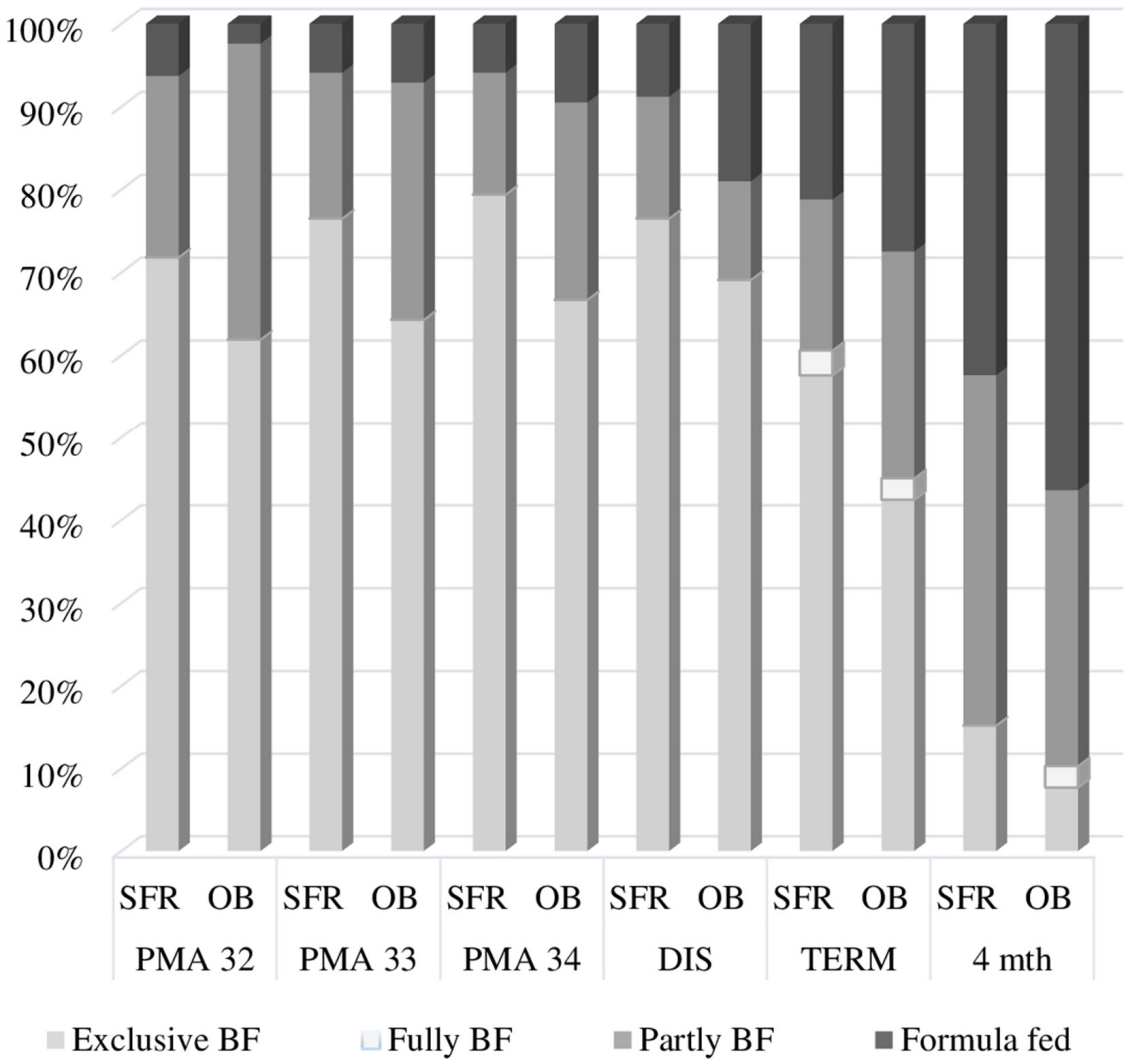
The Extent to Which Infants Received Mother’s Milk. *Note*. The percentage distribution of the extent to which infants received mother’s milk in the single-family room unit (SFR) and the open bay unit (OB) at infants’ post-menstrual age (PMA) 32, 33, and 34 weeks, at discharge, term date, and 4 months corrected age, defined in line with WHO criteria as: Exclusively Breastfed ([BF] drops, syrups, medicine), Fully BF (liquids, drops, syrups, but not non-human milk or food based fluids), Partly BF (mother’s milk supplemented with other nutrition), or Formula-Fed (WHO, 2019).

The first time milk was expressed differed, with median hours 24 hr earlier in the SFR unit compared to the OB unit (6 [6–11] vs. 30 [27–40], *p* = < .001). Neither volumes of milk at Days 7, 14, and PMA 34^0^ (Table 3), or the adjusted mean difference in volumes of mother’s milk; *B* = 41 ml (95% CI [−33.0, 314.3]), differed significantly between the units, but participant mothers of twins produced significantly more milk than participant mothers of singletons; *B* = 233 ml (95% CI [−356.6, −88.49]). In both units most participants established sufficiently large milk production to feed their infant(s) with their own milk exclusively or partly, and many still provided mother’s milk to some degree at 4 months’ corrected age (equivalent to 6–7 months’ chronological age; Figure 2). At discharge, the probability for participant infants being fed mother’s milk (exclusively and partly) differed in favor of the SFR unit (88.8% vs. 80.9% probability, respectively). To determine if there was an association between SFR care and the extent to which infants were fed mother’s milk exclusively from PMA 34 weeks until 4 months corrected age, a linear mixed model analysis was performed. We found the odds ratio for participant infants to be classified in the less exclusive categories fully, partly, or formula fed decreased by a factor of 0.4 with SFR care compared to OB care; Exp (B) = .4 (95% CI [.2, 1.0], *p* = .04). Thus, the likelihood for participant infants to be fed mothers milk more exclusively was increased with SFR care compared to OB care.

**Table 3:** Comparison of Infant Feeding Patterns Grouped by Type of Hospital Care (Infants *n* = 77; Mothers *n* = 66).

Variables	Type of Hospital Care				*t*	*p*
	SFR *M (SD*)	Min-Max	OB *M (SD*)	Min-Max		
Infants	*n* = 35 (45.5%)		*n* = 42 (54.5%)			
Total sessions at the breast	26 (16)	0–62	27 (16)	0–67	-.38	.71
Mothers	*n* = 30 (45.5%)		*n* = 36 (54.5%)			
BF self-efficacy	54 (13)	22–70	51 (13)	22–70	.75	.46
Total volume of milk produced ml/24 hr						
Day 7 post-delivery	543 (436)	24–1495	376 (297)	6–1090	1.91	.08
Day 14 post-delivery	660 (456)	0–1600	491 (381)	6–1640	1.71	.06
PMA 34°	686 (403)	0–1580	527 (334)	1–1250	1.79	.12

*Note*. SFR = single-family room unit; OB = open bay unit; BF = breastfeeding; PMA = post-menstrual age; ° = 0 days. Total sessions at the breast occurred during post-menstrual age 32°–34°; Breastfeeding self-efficacy was measured by the Breastfeeding Self-Efficacy Scale–Short Form (BSES–SF) questionnaire (Supplementary Files) administered at discharge to the remaining directly breastfeeding mothers *N* = 45 (58%), *n =* 25 SFR; *n* = 20 OB; Self-Efficacy score ranged from 14 to 70, higher score indicating higher level of self-efficacy. Volume of total milk produced = expressed and/or directly breastfed (measured with test weighing; 1 g of infant weight gain was considered equivalent to 1 mL of milk).

The time of first attempt at direct breastfeeding differed, with median hours 61 hr earlier in the SFR unit (48 [47–100] vs. 109 [96–183], *p* = < .001). Neither the number of breastfeeding sessions, the BSES–SF score (Table 3) nor the use of nipple shields differed between the units, and most participant infants in both units were directly breastfed to some degree at discharge, 80% (*n* = 28) in the SFR unit and 76% (*n* = 32) in the OB unit respectively (Table 4). However, significantly more participant infants were exclusively directly breastfed in the SFR unit compared to participant infants in the OB unit at discharge. In fact, all participant infants in the SFR unit who were exclusively fed their mother’s own milk were also exclusively directly breastfed, whereas most participant infants in the OB unit were categorized as partly directly breastfed as they were fed their mother’s expressed breastmilk by bottle in addition to directly breastfeeding (Table 4). For every participant infant classified as exclusively directly breastfed at discharge with OB care, there were seven with the same classification with SFR care. At discharge, the probability that exclusively directly breastfeeding would occur was 56.2 percentage points higher with SFR care compared to OB care (65.7% vs. 9.5% probability, respectively). The difference was less pronounced at term and 4 months corrected age (30.9 and 4.3 percentage points respectively). To measure the strength of the association between SFR care and occurrence of exclusively directly breastfeeding from discharge until 4 months corrected age, a logistic mixed model analysis was performed. The adjusted odds ratio for participant infants to sustain exclusively directly breastfeeding from discharge until 4 months corrected age was increased by a factor of 6.8 with SFR care compared to OB care; *OR* = 6.8 (95% CI [2.4, 19.1], *p* = < .001), indicating that exclusively directly breastfeeding is more likely to occur with SFR care.

**Table 4:** Comparison of Direct Breastfeeding and Use of Nipple Shields Between Type of Hospital of Care (infants *N*= 77).

Variable	Hospital Care		*Χ* ^2^	*p*
	SFR *n* = 35 (45.5%) *n* (%)	OB *n* = 42 (54.5%) *n* (%)		
At discharge				
Exclusively directly BF	22 (63)	4 (10)	22.8	< .001
Partly directly BF^a^	6 (17)	28 (66)	16.06	< .001
Not directly BF	7 (20)	10 (24)	0.52	.32
At term				
Exclusively directly BF	20 (57)	11 (26)	5.21	.02
Partly directly BF^a^	3 (9)	17 (41)	8.58	.003
Not directly BF	12 (34)	14 (33)	0.08	.78
At four months corrected age				
Exclusively directly BF	4 (11)	3 (7)	0.44	.51
Partly directly BF^a^	13 (38)	12 (29)	0.00	.00
Not directly BF	18 (51)	27 (64)	1.00	.32
Introduced to solids	27 (77)	36 (86)	1.11	.29
Nipple shields				
PMA 32 weeks	4 (13)	6 (14)	0.00	.00
PMA 33 weeks	7 (21)	14 (33)	0.96	.33
PMA 34 weeks	15 (44)	15 (36)	0.26	.61
At discharge	10 (30)	12 (29)	0.00	.00
At term	5 (16)	6 (15)	0.00	.00
At 4 months CA	0 (0)	0 (0)	-	-

*Note*. SFR = single-family room unit; OB = open bay unit; BF = breastfed; PMA = post-menstrual age; CA = corrected age. ^a^Partly directly fed infants were also fed by bottle.

## Discussion

We found hospitalization in a SFR NICU associated with earlier initiation of expressing milk and breastfeeding attempts, and participant infants were fed mothers milk and exclusively directly breastfed to a greater extent until 4 months corrected age. Despite a positive trend in favor of the SFR unit regarding breastfeeding self-efficacy (BSE) and milk volumes produced, the differences did not reach significance.

Previously, researchers studying breastfeeding in SFR NICUs have mainly focused on the volumes of mother’s milk produced or the extent to which infants are fed human milk, rather than on direct breastfeeding. In a systematic review and meta-analysis, van Veenendaal et al. (2019) found a higher instance of exclusive breastfeeding at discharge in SFR care compared to OB care, applying a definition of breastfeeding as “receiving the mother’s milk.” In a comprehensive study on SFR design, Lester et al. (2016) reported higher levels of “feeding” (but not direct breastfeeding) in the SFR unit as part of their maternal involvement outcome. From the same cohort, Vohr et al. (2017) reported higher volumes of mothers’ milk produced and human milk intake in the SFR unit.

In this study, most participant mothers initiated milk expression and direct breastfeeding, with no significant difference between units regarding BSE. To our knowledge, this study was the first to report on the timing of first expression and first attempt of direct breastfeeding, and to compare BSE in the SFR context. We know of no other studies concerning use of the SFR design providing breastfeeding data until 4 months corrected age. In OB units, provision of the mother’s own milk and breastfeeding as much and as often as possible can become even more important, and may somewhat compensate for the separation caused by the lack of optimized NICU facilities (Flacking et al., 2016). Whereas the SFR design offers unlimited presence and privacy, mothers in OB units are constantly surrounded by staff and other mothers in a similar situation. This may affect BSE through observational learning, role modeling and verbal persuasion (Brockway et al., 2017). This may ameliorate the disadvantages of not having an optimized physical environment in OB units.

Although participant mothers in the SFR unit initiated milk expression and breastfeeding attempts earlier, and subsequently attained exclusively direct breastfeeding more often than participant mothers in the OB unit did, we could not demonstrate that SFR care was associated with increased volumes of mother’s milk produced, which contradicts other researchers (Vohr et al., 2017). Even so, the adjusted mean differences are rather large, and may therefore be considered clinically relevant for the infants in this sample.

Maintenance of a sufficient milk supply until the infant is able to breastfeed directly is a prerequisite to feeding the infant exclusively with their mother’s own milk. Given the lack of differences in volumes of milk produced and BSE, we find it probable that the significantly higher likelihood for attaining exclusive direct breastfeeding was related to the facilitation of continuous mother–infant closeness in the SFR unit. Continuous maternal presence is indeed fundamental in order to attain exclusive direct breastfeeding. The SFR design allowed mothers to be present around the clock, provide SSC, express milk, and breastfeed in privacy whenever they wanted, including during the night. In OB units, mothers are visitors and spend many hours every day away from their infant. Thus, infants had to be fed by staff in the participating OB unit, by gavage or cup, until direct breastfeeding was considered established; and thereafter by bottle if the mothers agreed to this. At discharge, most participant mothers in the OB unit combined direct breastfeeding with expressing milk for feeding in their absence, as the OB design limited their ability to breastfeed around the clock. Notably, these feeding patterns were generally maintained after discharge from both units; very few participant mothers in the OB unit attained exclusive direct breastfeeding after leaving the hospital.

Our study may have been underpowered to detect a statistically significant difference in volumes of milk or BSE. On the other hand, a lack of difference may also be due to the positive general attitude towards breastfeeding in Norway. Cultural expectations, verbal persuasion, and support may enhance maternal efforts to accomplish breastfeeding (Brockway et al., 2017). The optimal duration of maternal presence or SSC needed to increase feeding with mother’s own milk or the occurrence of direct breastfeeding is not known. There is, however, convincing evidence that maternal presence, involvement in care, and SSC mediate infant outcomes, and that early initiation of SSC most likely triggered a cascade of maternal involvement, including breastfeeding (Lester et al., 2016). In both units, the levels of participant maternal presence were much higher than the maternal involvement reported by Lester et al. (2016) and several hours of SSC were obtained in both units on a daily basis.

Also fathers are important supporters of mothers who express milk and breastfeed after a preterm birth (Denoual et al., 2016). In Norway, infants have the right to have both parents present during hospitalization with full economic compensation for loss of income, and parental leave is compensated throughout the infant’s first year. This may facilitate breastfeeding. The positive cultural attitude towards breastfeeding, the observed high prevalence of breastfeeding initialization, and the high volumes of mothers’ milk produced and fed to infants in both units may differ from the base levels of other studies.

This study was conducted before the COVID pandemic. Upon submission, the infection rate in Norway was still very low and neither maternal presence nor breastfeeding had been restricted in healthy mothers. Still, public health measures put in place did limit parental presence by allowing only the mother or only one parent at a time in the NICUs. The consequences of these restrictions are unknown and pose a risk of potentially negative consequences. On a general note, one could argue that any public health measures put into place during the pandemic restricting parental presence or participation in infant care poses a risk to the principles of family-centered care and the parent–infant dyad.

NICU design and care culture are important for facilitating continuous maternal presence with increased mother–infant physical closeness. Because little is known about the optimal level of parental presence or SSC for milk production and breastfeeding, how breastfeeding support in SFR units should be delivered in order to improve maternal BSE, milk production, and direct breastfeeding after a preterm birth, or how public health measures put into place during the pandemic affects family-centered care and the parent–infant dyad, further research is required.

### Limitations

For some of our outcomes, the study may have been underpowered to detect a statistically significant difference between the two units. Furthermore, cultural differences or care practices (i.e., staff attitudes and breastfeeding guidance) and the need for transport by ambulance to the OB NICU may have influenced initiation of expressing and first breastfeeding attempt. Even so, we have controlled for many factors affecting breastfeeding.

## Conclusion

Optimizing nutrition with mother’s milk for the preterm population is a central WHO strategy to improve infant outcomes after a preterm birth. We demonstrated that a high degree of mother’s milk nutrition was achievable after a preterm birth, that a SFR NICU design allowing maternal presence around the clock was associated with earlier initialization of expression and attempts at breastfeeding, and that infants were fed mothers’ milk and exclusively directly breastfed to 4 months corrected age to a greater extent with SFR NICU care. The SFR design did not improve maternal BSE or volumes of milk produced.

## Supplemental Material

Supplementary Material 1 - Supplemental material for Associations Between Single-Family Room Care and Breastfeeding Rates in Preterm Infants Click here for additional data file. Supplemental material, Supplementary Material 1, for Associations Between Single-Family Room Care and Breastfeeding Rates in Preterm Infants by Hege Grundt, Bente Silnes Tandberg, Renée Flacking, Jorunn Drageset and Atle Moen in Journal of Human Lactation
