# Supplementary material for: Associations Between Single-Family Room Care and Breastfeeding Rates in Preterm Infants
Source: J Hum Lact. 2020 Oct 9;37(3):593–602. doi: 10.1177/0890334420962709 (PMC8414820; doi:10.1177/0890334420962709)
Supplement: Supplementary Material 1 - Supplemental material for Associations Between Single-Family Room Care and Breastfeeding Rates in Preterm Infants [file 10.1177_0890334420962709-suppl1.pdf]

Breastfeeding Self-Efficacy ID: __________

This questionnaire is to be answered by mothers who directly breastfeed at discharge from the NICU. Below are allegations of breastfeeding. Please indicate the extent to which you agree or disagree

	Rarely	Sometimes	Regularly	Often	Always
I can always determine that my baby is getting enough milk	1	2	3	4	5
I can always successfully cope with breastfeeding like I have with other challenging tasks	1	2	3	4	5
I can always ensure that my baby is properly latched on for the whole feeding	1	2	3	4	5
I can always breastfeed my baby without using formula as supplement	1	2	3	4	5
I can always manage the breastfeeding situation to my satisfaction	1	2	3	4	5
I can always manage to breastfeed even if my baby is crying	1	2	3	4	5
I can always keep wanting to breastfeed	1	2	3	4	5
I can always comfortably breastfeed with my family members present	1	2	3	4	5
I can always be satisfied with my breastfeeding experience	1	2	3	4	5
I can always deal with the fact that breastfeeding can be time consuming	1	2	3	4	5
I can always finish feeding my baby on one breast before switching to the other breast	1	2	3	4	5
I can always continue to breastfeed my baby for every feeding	1	2	3	4	5
I can always manage to keep up with my baby's breastfeeding demands	1	2	3	4	5
I can always tell when my baby is finished breastfeeding	1	2	3	4	5

Thank You.
